# Genotypic and phenotypic correlations of biotinidase deficiency in the Chinese population

**DOI:** 10.1186/s13023-018-0992-2

**Published:** 2019-01-07

**Authors:** Rai-Hseng Hsu, Yin-Hsiu Chien, Wuh-Liang Hwu, I-Fan Chang, Hui-Chen Ho, Shi-Ping Chou, Tzu-Ming Huang, Ni-Chung Lee

**Affiliations:** 1 Department of Medical Genetics, National Taiwan University Hospital, No. 8, Chung-Shan S. Rd., Zhongzheng Dist., Taipei, 10041 Taiwan; 2 Department of Pediatrics, National Taiwan University Hospital, No. 8, Chung-Shan S. Rd., Zhongzheng Dist., Taipei, 10041 Taiwan; 3 Department of Pediatrics, Taipei Medical University Hospital, No. 252, Wuxing St, Xinyi Dist., Taipei, 11031 Taiwan; 4 Taipei Institute of Pathology, No.146, Sec.3, Chongqing N. Rd., Datong Dist., Taipei, 10374 Taiwan

**Keywords:** Biotinidase deficiency, Chinese population, Newborn screening program

## Abstract

Biotinidase deficiency is an autosomal recessive disorder that affects the endogenous recycling and release of biotin from dietary protein. This disease was thought to be rare in East Asia. In this report, we delineate the phenotype of biotinidase deficiency in our cohort. The genotypes and phenotypes of patients diagnosed with biotinidase deficiency from a medical center were reviewed. The clinical manifestations, laboratory findings, and molecular test results were retrospectively analyzed. A total of 6 patients were evaluated. Three patients (50%) were diagnosed because of a clinical illness, and the other three (50%) were identified by newborn screening. In all patients, the molecular results confirmed the *BTD* mutation. The three patients with clinical manifestations had an onset of seizure at the age of 2 to 3 months. Two patients had respiratory problems (one with apnea under bilevel positive airway pressure (BiPAP) therapy at night, and the other with laryngomalacia). Hearing loss and eye problems were found in one patient. Interestingly, cutaneous manifestations including skin eczema, alopecia, and recurrent fungal infection were less commonly seen compared to cases in the literature. None of the patients identified by the newborn screening program developed symptoms. Our findings highlight differences in the genotype and phenotype compared with those in Western countries. Patients with biotinidase deficiency benefit from newborn screening programs for early detection and management.

## Introduction

Biotinidase deficiency (MIM #253260; BTD) is an autosomal recessive disorder affecting the endogenous recycling and release of biotin from dietary protein [1]. BTD results in low activities of biotin-dependent carboxylases and urinary excretion of organic acids characteristic of multiple carboxylase deficiency (MCD). BTD was first known as late-onset MCD because most patients present first symptoms after a month of age [2], and in 1982, Wolf et al. found that biotinidase is the primary enzymatic defect in late-onset MCD [3]. Patients with BTD can be divided into profound (residual activity < 10%) and partial deficiency (10–30%) due to the biphasic distribution of residual enzyme activity [4]. Patients with profound BTD manifest with cutaneous symptoms including dermatitis, conjunctivitis, and alopecia and neurological symptoms including hypotonia, seizures, developmental delay, hearing loss [5], and optic atrophy [6] at an early age. Affected patients, if left untreated, can progress to metabolic decompensation, coma, or death [7]. Patients with partial BTD usually have milder symptoms during stress at a later stage, and the disease can remain undiagnosed in children presenting developmental delay [8] or autism [9]. Symptoms of biotinidase deficiency can be prevented by therapeutic doses of biotin (5–20 mg daily) [10, 11], but neurological deficits are not reversible once they occur [10, 12].

Newborn screening for BTD can be conducted by determination of biotinidase activity on dried blood spots (DBS) [13]. Currently, all newborn screening programs in the United States and more than 30 other countries screen for BTD [14]. Patients diagnosed by newborn screening and treated with biotin before symptom onset develop normally. The incidence of BTD is approximately 1/60,000 (approximately 1/120,000 for both the partial and profound types) according to one early study of worldwide newborn screening for biotinidase deficiency [15]. The incidence varies between countries, and higher incidences were noted in Brazil, Turkey and Saudi Arabia [16–18].

BTD was thought to be rare in East Asia. Four patients with MCD were identified from 606,380 newborns in a pilot newborn screening program in Japan [19]. In a report from China, 4 patients with BTD were identified by selective screening of 9100 patients with suspected inborn errors of metabolism, but no cases were detected by screening 116,000 newborns [20]. One pilot screening program in Taiwan failed to detect any patients among 60,000 newborns (personal communication with Taiwan Institute of Pathology). In this study, we performed a retrospective review of BTD from a medical center. We identified 6 patients with this disease, and our data suggest that BTD still causes significant morbidity in our cohort.

## Methods

### Patients

Hospital medical history databanks from January 2003 to September 2016 with six BTD patients were retrospectively analyzed. The study protocol was approved by the Institutional Review Board of our hospital (No. 201612034RIND). Items of clinical manifestations that were collected included initial symptoms, presence of seizure, growth and development, skin manifestations including eczema and alopecia, and family history. Laboratory data including acylcarnitine profiles, urine organic acid analysis, biotinidase activity, and BTD mutations were also collected.

### Biotinidase activity and *BTD* mutation analysis

Biotinidase activity was measured by fluorescence assays using dried blood spots (DBS) (Neonatal Biotinidase kit, PerkinElmer®, Wallac Oy, Turku, Finland). The mean activity from newborns was 160.1 ± 39.93 nmol/min/dL. *BTD* mutation analysis was performed by Sanger sequencing using NM_000060.2 and NP_000051.1 as references. Variations detected by sequencing were annotated by ClinVar (https://www.ncbi.nlm.nih.gov/clinvar/) and HGMD (The Human Gene Mutation Database, http://www.hgmd.cf.ac.uk/ac/index.php) and searched in the *BTD* database from Department of Pathology, the University of Utah (http://www.arup.utah.edu/database/BTD/BTD_welcome.php).

## Results

From January 2003 to September 2016, 6 patients were diagnosed with BTD in our hospital (Table 1). They were all born to Chinese parents except that the father of patient 5 is an Indian. Three patients were diagnosed because of clinical illness, and the other three were identified by newborn screening.

**Table 1:** Finding in patients with biotinidase deficiency

Patient	1	2	3	4	5	6
Diagnosed by	Clinical	Clinical	Clinical	Newborn screen	Newborn screen	Newborn screen
Biotinidase activity (nmol/min/dL) (% of control mean)	12.5 (7.8%)	15.4 (9.8%)	NA	36.5 (23%)	36.3 (23%)	32.5 (20%)
C5OH at newborn screening (μM)	NA	0.17	NA	0.14	0.10	0.12
C5OH at presentation (μM)	3.5	3.37	0.362	–	–	–
BTD mutations	c.460-1G > A/c.1382 T > C (p.V461D)	c.460-1G > A/c.1382 T > C (p.V461D)	c.1384delA (p.R462Gfs) homozygous	c.1250_1251TC > AG (p.V417E)/c.1306G > A (p.E436K)	c.1361A > G (p.Y454C)/c.1306G > A (p.E436K)	c.1250_1251TC > AG (p.V417E)*
Urine GCMS	Elevation of 3-hydroxyisovalerate, 3-methylcrotonylglycine, lactate, pyruvate	Elevation of 3-hydroxyisovaleric acid	Elevation of 3-hydroxyisovaleric acid	NA	NA	No specific finding
Seizure onset age	3 m	3 m	2 m	–	–	–
Respiratory problems	Apnea requiring BiPAP at night	Laryngomalacia	–	–	–	–
Hearing loss	+	–	+	–	–	–
Optic atrophy	+	–	NA	–	–	–
Eczema	+	+	NA	−	−	–
Alopecia	–	–	–	–	–	–
Candidiasis	–	Diaper rash	NA	–	–	–
Current status	10y, developmental delay	3y, normal development	Expired at 2y, developmental delay	5 m, asymptomatic	1 m, asymptomatic	NA

NA: not available, C5OH normal < 0.182 μM, *one allele deletion cannot be excluded

### Case description

Patient 1 was noted to have seizures and an elevated plasma lactate level (4.95 mM, normal < 2.2 mM) when she was 3 months old. At the age of 6 years, she could only sit with support, babbled, and had hearing impairment, optic nerve atrophy, sleep apnea, proximal type renal tubular acidosis, and seizures that were controlled by a ketogenic diet. A brain magnetic resonance imaging (MRI) study revealed diffuse high intensity of white matter on T2-weighted images and a decreased N-acetylaspartate-to-choline (NAA/CHO) ratio and presence of a lactate signal on magnetic resonance spectrometry (MRS). A muscle biopsy revealed abnormal mitochondria reminiscent of mitochondrial disease, but sequencing of mitochondrial DNA revealed no pathogenic variants. She had recurrent eczema-like skin lesions. Her correct diagnosis was made after the diagnosis of her younger brother. Currently she still had apnea and needed a bilevel positive airway pressure (BiPAP) respirator at night time. She also had hearing loss.

Patient 2, the younger brother of patient 1, had laryngomalacia and seizures at the age of 3 months after an episode of respiratory tract infection. At that time, generalized skin rash, hypotonia, stridor, and septic shock were noted. A brain MRS study revealed the presence of a lactate peak. MCD was then suspected because his DBS C5OH-carnitine level was elevated (3.37 μM, normal < 0.36 μM). In addition, low biotinidase activity (15.4 nmol/min/dL) was noted. After the diagnosis of BTD, biotin 5 mg/day was prescribed. Stabilization of vital signs, increased muscle tone and activity, and resolution of skin lesions were observed in one week. He is now 3 years old and is seizure-free with normal development. His sister, at 8 years of age, started to learn to walk and had verbal interactions with family members after biotin treatment for approximately 1.5 years.

Patient 3 had seizures since 2 months of age. She could not sit or turn over and had poor responses to stimuli at the age of 2 years, when she died. Her DBS C5OH-carnitine level was mildly elevated (0.362 μM, normal < 0.182 μM), but urine organic acid analysis revealed an elevation of 3-OH-isovaleric acid levels. 3-Methylcrotonyl-CoA carboxylase deficiency was suspected, but her symptoms were not relieved after leucine restriction and carnitine supplementation. She died at 2 years of age. Final diagnosis was made after death.

Patients 4, 5 and 6 were found to have low DBS biotinidase by newborn screening after screening 46,958 newborns in an 11-month period in our screening center. Biotin 5 mg/day has been prescribed for patient 4. Patients 4, 5 and 6 are currently asymptomatic.

### Biochemical profiles

General laboratory tests are not informative in BTD. Elevation of lactic acid may be a clue for metabolic diseases. This finding was described in patient 1, who had a plasma lactate level of 4.95 mM (normal < 2.2 mM) when she was 3 months of age, and in patient 2 because of a lactate peak identified by a brain MRS study. Elevation of C5OH-carnitine is not a reliable biomarker for biotinidase deficiency. C5OH-carnitine levels were significantly elevated in patient 1 (3.53 μM, normal < 0.74 μM) at 6 years of age and in patient 2 (3.37 μM) at the age of 3 months. However, the C5OH-carnitine level was normal in patient 2 at birth by newborn screening and was only mildly elevated in patient 3 (0.362 μM) at the age of 2 years. Abnormal results of urine organic acid analysis were reported for patients 1–3 as an elevation of 3-OH-isovaleric acid levels with or without other characteristic abnormal organic acids in urine, including 3-methylcrotonylglycine, lactate and pyruvate, suggesting MCD.

### Biotinidase activity

Biotinidase activities in the first two patients were very low: 15.4 nmol/min/dL (9.8% of control mean) and 12.5 nmol/min/dL (7.8% of control mean). However, the activities in the three patients detected by newborn screening were higher: 36.5 nmol/min/dL (23% of control mean), 36.3 nmol/min/dL (23% of control mean), 32.5 nmol/min/dL (20% of control mean), respectively.

### Molecular analysis

Patients 1 and 2 had compound heterozygous mutations c.460-1G > T and c.1382 T > A (p.V461D) on the BTD gene, which were inherited from both parents. Mutation analysis after the death of patient 3 revealed a homozygous c.1384delA variant of the BTD gene. Patient 4 had compound heterozygous c.1250_1251TC > AG (p.V417E) and c.1306G > A (p.E436K) mutations. Patient 5 had c.1361A > G (p.Y454C)/ c.1306G > A (p.E436K) mutations. Patient 6 had c.1250_1251TC > AG (p.V417E) homozygous mutation or deletion. Two of the variants, c.1384delA and c.1361A > G, had been reported: the patient with homozygous c.1384delA had 13% biotinidase activity but suffered from apnea, unconsciousness, convulsions, hearing impairment, and mental retardation [20]; the patient with compound heterozygous c.1361A > G mutation had profound biotinidase deficiency [21]. The other four, c.460-1G > T, c.1382 T > A, c.1250_1251TC > AG, and c.1306G > A, are novel variants but are predicted as pathogenic or likely pathogenic (Table 2).

**Table 2:** List of mutations identified in the current study

BTD mutation	Physical position (Hg19)	dbSNP	ARUP classification	ClinVar	HGMD	MAF	gnomAD total frequency	TW biobank	SIFT	Polyphen − 2	ACMG criteria
c.460-1G > A	chr3:15685822	NA	NA	NA	NA	NA	NA	NA	NA	NA	Pathogenic (PVS1 PM2 PM3)
c.1250_1251TC > AG (p.V417E)	chr3:15686613-chr3:15686614	rs750006399 / rs764811197	NA	NA	NA	0.0002718 (all East Asian)	0.00001989	0.000989	D	PD	Likely pathogenic (PM2 PM3 PP3 PP4)
c.1306G > A (p.E436K)	chr3:15686669	rs749460715	NA	Likely pathogenic / VUS	NA	0.0004510 (all East Asian)	0.00003182	0.000989	D	PD	Likely pathogenic (PM2 PM3 PP3 PP5)
c.1361A > G (p.Y454C)	chr3:15686724	rs397514345	Pathogenic	Likely pathogenic / Pathogenic / VUS	DM	0.002058 (all South Asian)	0.0002625	NA	D	PD	Likely pathogenic (PM2 PM3 PM5 PP3 PP5)
c.1382 T > C (p.V461D)	chr3:15686745	NA	NA	NA	NA	NA	NA	NA	T	B	Likely Pathogenic (PM2 PM3 PP3 PP4)
c.1384delA (p.R462Gfs)	chr3:15686747–15,686,747	rs397514420	Pathogenic	Pathogenic	DM	0.0001087 (all East Asian)	0.000007955	NA	NA	NA	Likely Pathogenic (PVS1 PM2 PM3 PP4 PP5)

NA, not found form the searched database, MAF, maximal minor allele frequency in gnomAD; D, deleterious; PD, probably damaging; VUS, uncertain significance; TW biobank, Taiwan biobank; DM, disease-causing mutation; T, tolerated; B, benign

## Discussion

In this paper, we described 6 patients with BTD in our cohort. Three patients (50%) were identified by neonatal screening, corresponding to an incidence of one in 15,653 in our cohort. None of those diagnosed by newborn screening had clinical manifestations suggestive of BTD. The other three patients (50%) were diagnosed based on clinical suspicion. The age at onset of clinical symptoms ranged from 2 to 3 months. Two of the three aforementioned patients had respiratory problems (one with apnea under BiPAP therapy at night, and the other with laryngomalacia). Hearing loss and optic atrophy were found only in patient 1.

Interestingly, cutaneous manifestations including skin eczema, alopecia, and recurrent fungal infection were less commonly seen compared with cases in the literature. In previous cohorts from Iran and India, alopecia was the common manifestation (8 of 16 and 9 of 10, respectively) [22, 23]. The first two patients had serum biotinidase activity < 10% of the control mean (classified as profound BTD), their mutations, c.460-1G > A/c.1382 T > C, though novel, must be severe. The early death of the third patients suggests profound BTD which is compatible with her homozygous null mutation c.1384delA.The first two patients with profound biotinidase deficiency were noted with eczema-like skin presentations but there was no alopecia. In the United States, the four mutations most commonly associated with complete biotinidase deficiency are C33Ffs*36, Q456H, R538C, and the double mutation D444H:A171T. Partial BTD is almost universally attributed to the D444H mutation [24, 25]. However, there is still a disparity between the genotype and biochemical phenotype of BTD, and biotinidase activity may be affected by both genetic and non-genetic factors (including age, prematurity, and neonatal jaundice) [26]. The genotypes in this study (Table 1) were all different from the above mentioned variants identified in the United States.

Newborn screening in Taiwan, started in 1981 [27], is partially reimbursed by the government and not mandatory but still covers 95–99% of newborns with not only the classic screening items but also a number of lysosomal storage diseases [28]. In comparison, newborn screening in the United State is mandatory but the programs vary between the states [29]. Screening for biotinidase deficiency was not considered in Taiwan because the disease was thought to be extremely rare. However, long-term experiences of biotinidase deficiency screening reveals low false positive rate and full prevention of clinical symptoms in all detected patients by early institution of biotin therapy [30]. Therefore, we started the screening in 2015, and currently our false positive rate with a cut-off of 35% of normal mean was lower than 0.03%. There are several economic evaluation methods that can be applied to newborn screening programs [31]. Vallejo-Torres et al. found that newborn screening for biotinidase deficiency led to higher quality quality-adjusted life years, and the probability that biotinidase deficiency screening was cost-effective was estimated to be > 70% [32]. We believe that newborn screening for biotinidase in Taiwan will also be cost effective. Recently, multiplex tandem mass spectrometry assay for newborn screening has include biotinidase deficiency which will make screening for this disease cheaper and more convenient [33].

## Abbreviations

BiPAP: Bilevel positive airway pressure
BTD: Biotinidase deficiency
DBS: Dried blood spot
MCD: Multiple carboxylase deficiency
MRI: Magnetic resonance imaging
MRS: Magnetic resonance spectrometry
